# How should social mixing be measured: comparing web-based survey and sensor-based methods

**DOI:** 10.1186/1471-2334-14-136

**Published:** 2014-03-10

**Authors:** Timo Smieszek, Victoria C Barclay, Indulaxmi Seeni, Jeanette J Rainey, Hongjiang Gao, Amra Uzicanin, Marcel Salathé

**Affiliations:** 1 Center for Infectious Disease Dynamics, Department of Biology, The Pennsylvania State University, University Park, PA 16802, USA; 2 Division of Global Migration and Quarantine, Centers for Disease Control and Prevention, 1600 Clifton Rd. MS-03, Atlanta, GA 30333, USA; 3 Current address: Modelling and Economics Unit, Public Health England, London NW9 5EQ, UK

**Keywords:** Contact networks, Social network, Proximity network, Droplet transmission, Contact survey, Wireless sensor network

## Abstract

**Background:**

Contact surveys and diaries have conventionally been used to measure contact networks in different settings for elucidating infectious disease transmission dynamics of respiratory infections. More recently, technological advances have permitted the use of wireless sensor devices, which can be worn by individuals interacting in a particular social context to record high resolution mixing patterns. To date, a direct comparison of these two different methods for collecting contact data has not been performed.

**Methods:**

We studied the contact network at a United States high school in the spring of 2012. All school members (i.e., students, teachers, and other staff) were invited to wear wireless sensor devices for a single school day, and asked to remember and report the name and duration of all of their close proximity conversational contacts for that day in an online contact survey. We compared the two methods in terms of the resulting network densities, nodal degrees, and degree distributions. We also assessed the correspondence between the methods at the dyadic and individual levels.

**Results:**

We found limited congruence in recorded contact data between the online contact survey and wireless sensors. In particular, there was only negligible correlation between the two methods for nodal degree, and the degree distribution differed substantially between both methods. We found that survey underreporting was a significant source of the difference between the two methods, and that this difference could be improved by excluding individuals who reported only a few contact partners. Additionally, survey reporting was more accurate for contacts of longer duration, and very inaccurate for contacts of shorter duration. Finally, female participants tended to report more accurately than male participants.

**Conclusions:**

Online contact surveys and wireless sensor devices collected incongruent network data from an identical setting. This finding suggests that these two methods cannot be used interchangeably for informing models of infectious disease dynamics.

## Background

Contact network data are useful for parameterizing models that aim to understand and predict infectious disease dynamics. For example, the dynamics of sexually transmitted diseases [1-5] and droplet transmitted diseases [6-10] in humans, in addition to various infectious diseases of livestock and wild animals [11-15], are all better understood because of monitoring and modeling the interaction between the respective hosts.

Various network statistics can be computed to investigate how disease might spread across networks, some of which correlate very well with the behavior of host-pathogen systems. For instance, individuals who have many different contact partners (also known as degree) per period of time are more likely to become infected, to infect others, and to become infected earlier during an epidemic than individuals with fewer contact partners [5,16,17]. Moreover, even pathogens with low infectiousness can cause epidemics in networks with a highly disperse degree distribution, as opposed to - otherwise similar - networks with a narrow degree distribution [18]. In highly clustered contact networks, the local depletion of hosts results in decreased disease spread [19,20], and disease incidence growth is polynomial rather than exponential, as observed in unclustered networks [21]. Finally, in networks with a very strong community structure (i.e., networks that can be easily separated into densely connected groups), individuals that connect different communities play a more important role for infectious disease spread than highly connected individuals within communities [22].

Several methods have been employed for measuring epidemiologically relevant contact data of host populations, including direct observations, videotaping, contact diaries and surveys, as well as wireless sensor network technologies [23]. In this paper, we focus on contact surveys and wireless sensor networks, both of which capture contacts that are sufficient for droplet transmission. Contact surveys for droplet transmitted infections were first introduced in 1997 [24], and have become a popular method for measuring the structure of potentially contagious contacts [25-30]. In contact surveys or contact diaries, study participants attempt to recall all contacts which meet a given definition and record these contacts on a paper or web-based survey form. More recently, wireless sensor networks have become available as a method for measuring contact networks [8,31-35]. Currently, these sensors all use similar technologies, are small, and can be easily worn by persons of all ages. The devices emit electromagnetic signals, which are detected and recorded by other sensors within a predefined distance.

The merits and drawbacks of various methods of contact network data collection, including surveys and sensors, have recently been reviewed [23]. Furthermore, several methodological contributions have assessed the features of the survey method. For example, one study compared retrospective and prospective survey designs [28], another study compared a web-based mode of data collection with a paper-based one [36], and a third study compared paper-based diaries with data collection on personal digital assistants (PDAs) [37]. Additionally, a recent study assessed reporting errors and biases in contact survey studies [38].

While both contact surveys and wireless sensor networks have their own particular strengths and weaknesses, a direct empirical comparison to understand whether these frequently applied methods can be used interchangeably has not yet been done. Here, we present the first study to our knowledge that compares contact surveys and wireless sensor networks as methods for collecting contact network data during a single school day at a United States (US) high school.

## Methods

### Ethics statement

This study was part of a bigger project [39]. The Pennsylvania State University Institutional Review Board (IRB) approved this project (IRB #37640), and the project was also approved under the US Centers for Disease Control and Prevention (CDC) IRB authorization agreement.

The project consisted of two parts, (1) a survey and wireless sensor network part (here) as well as (2) a disease surveillance part (including swabbing; data not used in this paper). Informed consent was obtained from all participants for the survey and wireless sensor network part, and all personal identifying information was removed after contact partner reports were matched with sensor IDs. Additionally, parental consent was obtained for the disease surveillance part for all participants younger than 18.

### Data collection

#### Setting

The contact network study was carried out at a US high school in the spring of 2012. When the data were collected, the entire school population was 974, which included 715 students as well as 259 teachers and other staff. The school was a full-time school and school days had between four and seven periods of 50 or 75 minutes duration. The student population was split in two halves for lunch break, one half having an early, one having a late break. Further, there were time slots assigned for school meetings, advisor meeting, and school clubs.

We did not offer individual incentives to participating students, teachers, or other staff. We did, however, involve the school community by offering scientific projects related to the study, in which students could participate and gain new skills.

#### Wireless sensor network

Wearable wireless sensor devices, herein referred to as motes, store close proximity records (CPR), which are a detection events between two motes. We used TelosB motes that were programmed to detect each other if the distance between them was two meters or less and only during face-to-face interactions (see Figure S4 in [8]). A distance cut-off of two meters was used in previous contact studies [30,38], and it corresponds well with theoretical work on droplet travel distances [40]. The mote beacon frequency was set to three per minute (i.e., every 20 seconds).

We distributed motes to students, teachers, and other staff at the high school on three days in the spring of 2012 [39]. During each deployment day, motes were placed in a pouch attached to a lanyard, and worn around participants’ necks during the school day. Each mote was labeled with a unique identification (ID) number. During the first mote day, participants reported on a paper form their full names and provided an e-mail address if they wanted to complete a web-based contact survey. Because we were interested in directly comparing the contact data collected by the motes to self-reported contact survey data, we only used mote data retrieved from the third mote deployment day, Tuesday, March 13, 2012, which was the same day for which the study participants were asked to report contacts in the web-based survey.

CPRs are concordant, if two motes record each other’s signal at the same time. Discordance means that only one mote recorded another mote’s signal, but not vice versa. Discordances were resolved by imputing missing CPRs.

#### Contact survey

Following close collaboration with school administrators to assess the appropriate timing, we distributed the web-based contact survey to participants by email on Wednesday, March 14, one day after the third mote deployment day. The school population was repeatedly informed when the contact survey would be sent by email and what day participants would be requested to remember their contact partners.

The online survey asked participants to recall and report all contacts that they had while at school the previous day, Tuesday, March 13. A contact in the survey was defined as a person with whom the participant had one or more interactions that (i) were a maximum of two arms-lengths apart, (ii) more than a 10-word conversation, and (iii) occurred only while at school. The distance cut-off of two arms-lengths was chosen as an easier-to-use proxy for the two meters cut-off that was defined for the wireless sensor network.

Contacts were entered in rows that consisted of two free text fields, where participants could enter the first and last name of their contact partners (or only one of the two if the other was unknown), and radio buttons to report the approximate aggregate contact duration (choice of 5 options: less than 5 minutes, 5 to 15 minutes, 15 to 60 minutes, 1 to 4 hours, longer than 4 hours). Initially, there was one empty row for the first contact. Participants could generate an unlimited number of further rows by pressing a clearly visible button labeled “add another contact”. Reminder emails were sent four days later, before the survey closed on Sunday, March 18.

### Linking survey and sensor data

As described above, survey participants were requested to report the names of all contact partners among the school population in the web-based contact survey. These names had to be matched with names associated with sensor ID numbers to make the two sources of data comparable. To do this, we developed a partly automated computer program to match the survey contacts with a list that linked participant names to sensor IDs.

For every contact partner reported by a study participant in the survey, we assessed the similarity of the contact’s name to the names of all individuals who participated in the mote study. We calculated average similarity scores for the first and last names combined, as well as similarity scores for both first and last names separately. The similarity scores could take values between 0 (totally different) and 1 (perfect match) and were defined as (2×*M*)/*T*, where *T* is the total number of elements in both text sequences and *M* is the number of matches [41]. Similarity scores were calculated using the ratio() method of the SequenceMatcher class of the difflib Library for Python 2.7.3 that is included in the Enthought Python Distribution 7.3-1 (32 bit).

A reported name was automatically replaced by the sensor ID if a perfect match between a mote study participant and a reported contact partner was found. The name was also automatically replaced by the sensor ID if all three of the following conditions were fulfilled: (i) either the first or the last name was a perfect match; (ii) the other part (i.e., either first or last) of the name had to have a similarity score of at least 0.9; (iii) there was only one match with a maximal similarity score and no additional names had the same score. If a matching problem could not be solved automatically, the names were manually reconciled by study staff, who were provided with the reported name and (i) the five mote study participants’ names with the highest average similarity score, (ii) the five names with the highest last name similarity scores, and (iii) the five names with the highest first name similarity scores. Study staff could then decide if one of the provided names matched the reported name, if the reported name was clearly a person who did not participate in the mote study (recode value was 999 instead of an ID), or if the matching problem could not be solved unambiguously (recode value 888 instead of an ID).

To ensure that all personal identifying information was removed from the contact data, each individual was given a new randomly allocated ID number.

### Analyses

#### Nodal degree comparisons

We compared the nodal degree reported from each web-based contact survey (without correcting for discordant reports) to the nodal degree recorded by the mote of each respective participant. To visualize congruence and difference in individual nodal degrees, we used scatter plots that map the mote-based degree of each participant on to her/his survey-based degree. We rendered plots for (i) all contacts reported/measured, (ii) all contacts of more than 5 minutes in duration, and (iii) all contacts of more than 15 minutes in duration. To assess the association between survey- and mote-based degree, we calculated the Kendall’s Tau rank correlation coefficient and estimated the corresponding 2-sided p-value. Finally, we visualized degree distributions for both survey- and mote-based contact data with kernel density plots. All plots were rendered using ggplot2 (version 0.9.3) that we linked into our main code (written in Python) with rpy2 (version 2.3.1, R version 2.15.2).

#### Average reporting probabilities

Having both contact survey and mote data collected from exactly the same setting, we analyzed whether differences in the two methods were a result of (i) underreporting of survey participants, or (ii) differences in the contact definitions underlying the two methods.

We used a previously developed method [38] to estimate reporting probabilities from the web-based contact survey. Briefly, the method is based on the premises that each contact should ideally be reported by two individuals, and that concordant and discordant contact reports can be used to estimate reporting probabilities.

 There are three possible combinations of reports:(i) both participants reported contact with each other (the number of such reports is labeled $N_{1}^{\mathrm{ss}}$), (ii) contact was reported by only one of the two involved participants ($N_{2+3}^{\mathrm{ss}}$), and (iii) neither of the two involved participants reported the contact ($N_{4}^{\mathrm{ss}}$, which is unknown).

**Figure 1:**
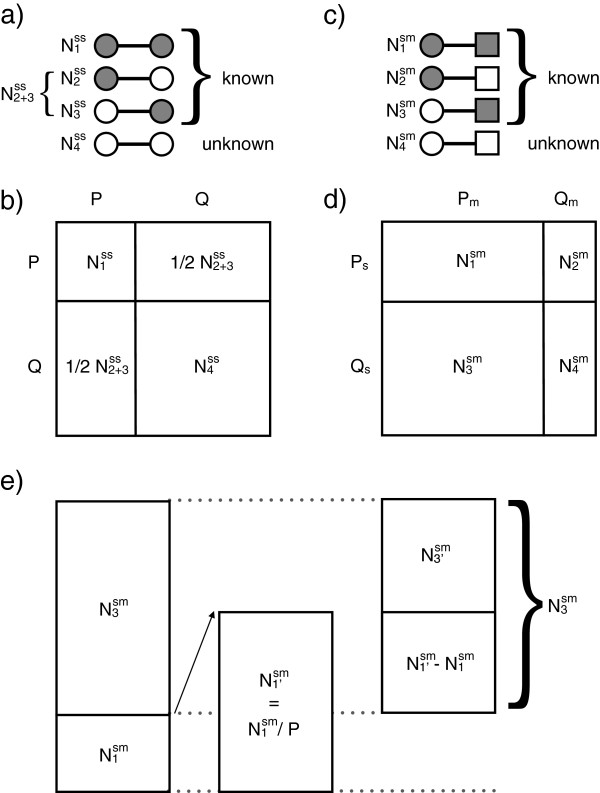
**Models of contact reporting probabilities. (a)** Contact reporting probabilities based on pairs of survey reports (^ss^), where a circle stands for a contact reported (grey) or not reported (white) by a survey participant. There are four possible combinations: (i) $N_{1}^{\mathrm{ss}}$ where both contact partners report the contact, (ii) $N_{2}^{\mathrm{ss}}$ and (iii) $N_{3}^{\mathrm{ss}}$ where only one of the contact partners report the contact (since $N_{2}^{\mathrm{ss}}$ and $N_{3}^{\mathrm{ss}}$ are indistinguishable, we use their sum $N_{2+3}^{\mathrm{ss}}$), and (iv) $N_{4}^{\mathrm{ss}}$ where none of the two contact partners report the contact. **(b)** All possible combinations of survey reporting statuses, where *P* stands for the probability of reporting a contact and *Q* is the complement. **(c)** Survey reports and mote detections of contacts combined (^sm^), where a rectangle stands for a contact detected (grey) or not detected (white) by a pair of motes. There are four possible combinations: (i) $N_{1}^{\mathrm{sm}}$ where a contact pair is reported in the survey and recorded by motes, (ii) $N_{2}^{\mathrm{sm}}$ and (iii) $N_{3}^{\mathrm{sm}}$ where only the survey or motes recorded the contact, and (iv) $N_{4}^{\mathrm{sm}}$ where a contact that actually took place was neither reported nor detected by a mote. **(d)***P*_*s*_ and *P*_*m*_ stand for average survey and mote reporting probabilities, and *Q*_*s*_ and *Q*_*m*_ are the average probability of a survey or mote not recording a contact respectively. **(e)** The estimated proportion of the difference between survey and mote data that can be attributed to survey underreporting, based on the models (b) and (d). Here, $N_{1'}^{\mathrm{sm}}$ is the estimated amount of contacts detected by the motes and also reported by the study participants if there was no underreporting (estimate is based on *P*), $N_{1'}^{\mathrm{sm}}\;-\;N_{1}^{\mathrm{sm}}$ is the estimated amount of mote-detected but not survey-reported contacts due to underreporting, and $N_{3'}^{\mathrm{sm}}$, is the estimated amount of non-reporting due to differences in contact definitions between the survey and mote studies.

The average probability estimate that a contact of a specific duration *t* is reported by a member of the population is then given by the equation $P\;=\;N_{1}^{\mathrm{ss}}\;/\;\left(N_{1}^{\mathrm{ss}}\;+\;\frac{1}{2}\;N_{2+3}^{\mathrm{ss}}\right)$. The complementary probability, *Q*, of not reporting a contact of duration *t* is *Q* = 1 - *P*. Figures 1a and b provide an illustration of how we calculated survey reporting probabilities. Probabilities were calculated for four types of contact durations: (a) 5 minutes or less; (b) 5 to 15 minutes; (c) 15 to 60 minutes; (d) 1 to 4 hours. Thereby, we assumed the higher contact duration value of every pair of contact reports to be true. Very few contacts lasted more than 4 hours; therefore, these contacts were excluded from the analysis.

We expanded on the method described above [38] by calculating differences between contact survey reporting and mote recordings. The probability that a contact is reported in the survey study, *P*_*s*_, conditional on being detected by the motes, *P*_*m*_, is defined as $P_{s}\;=\;N_{1}^{\mathrm{sm}}\;/\;\left(N_{1}^{\mathrm{sm}}\;+\;N_{3}^{\mathrm{sm}}\right)$. Survey reporting is expected to be incomplete and, hence, *P*_*s*_ < 1 and *Q*_*s*_ > 0, where *Q*_*s*_ is the probability of a contact detected by a mote but not reported in the survey. Figures 1c and d provide an illustration of the methods we used to calculate the probability that a mote detection is reported as a contact in the survey. Differences in reporting between the surveys and motes were calculated independently for each of the four contact duration categories described above. Thereby, we assumed the contact duration measured by the wireless sensor network to be true.

The non-reporting of contacts that were detected by the motes, represented by $N_{3}^{\mathrm{sm}}$, can have two causes: (i) underreporting by study participants (probability *Q*), or (ii) differences in the contact definitions underlying both the mote and the survey study. Regarding the latter: motes measure all face-to-face collocation events with a maximal distance of two meters between any two study participants, while the survey’s contact definition only includes conversational contact. Hence, even with perfect reporting, we would expect legitimately fewer survey contact reports than mote detections. Figure 1e illustrates the relationship between differences in both contact datasets (i) due to underreporting ($N_{1'}^{\mathrm{sm}}\;-\;N_{1}^{\mathrm{sm}}$), (ii) due to differences in contact definitions ($N_{3'}^{\mathrm{sm}}$), and relative to the total difference between both datasets ($N_{3}^{\mathrm{sm}}$).

The proportion of the difference between mote measurements and survey data that is caused by underreporting is given by $\left(N_{1'}^{\mathrm{sm}}\;-\;N_{1}^{\mathrm{sm}}\right)/\;N_{3}^{\mathrm{sm}}$; the proportion of the difference that is caused by differing contact definitions is given by $N_{3'}^{\mathrm{sm}}\;/\;N_{3}^{\mathrm{sm}}\;=\;1-\;\left[\left(N_{1'}^{\mathrm{sm}}\;-\;N_{1}^{\mathrm{sm}}\right)/\;N_{3}^{\mathrm{sm}}\right]$.

#### Individual differences in reporting quality

Individual differences in reporting probabilities cannot be estimated using only survey contact data (see supplementary material in [38]); however, our data allow us to calculate differences in individual reporting probabilities between contact survey data and the mote data, as we assumed that all motes record data with the same accuracy. To assess individual reporting differences, we determined individual *P*_*s*_ values for each participant and also for each of the four time duration categories. Individual differences in reporting quality could then be specifically attributed to differences at the individual level.

We assessed individual differences in reporting quality for all participants together, but also for groups defined by gender and by age. Differences between the mean individual reporting probabilities of these groups were tested for significance using a permutation test [42] with 99999 permutation resamples.

## Results

### Descriptive statistics

The mote derived contact network dataset covers CPRs from 487 (50.0%) individuals of the entire school population, and 320 (32.9%) of them also participated in the base survey, that asked questions about demographics and health. Two hundred and fifty six (26.3%) individuals participated in the contact survey and 245 (25.2%) reported at least one matchable contact in the survey. There were 178 participants (18.3%) for whom we had demographic information, at least one matchable contact report, and mote data. Of those, for whom we had all information, 109 (61.2%) were female and 69 (38.8%) were male. Furthermore, 138 (77.5%) were students and 40 (22.5%) were teachers or other staff.

Of the 1935 contact reports (i.e., directed ties) in the web-based survey, 253 (13.1%) were reported to have lasted less than 5 minutes, 507 (26.2%) were from 5 to 15 minutes, 986 (51.0%) were from 15 to 60 minutes, 179 (9.3%) were from 1 to 4 hours, and 10 (0.5%) were reported to have lasted more than 4 hours. Of these 1935 contact reports, 1475 (76.2%) could be clearly linked to mote study participants, 32 (1.7%) were not clearly identifiable and were given the ID value 888, and 428 (22.1%) referred to members of the school population who did not participate in the study. Three of the 32 not clearly identifiable names were from contacts with a duration of less than 5 minutes, 7 were from contacts with a duration between 5 and 15 minutes, 19 were from contacts with a duration between 15 and 60 minutes, and 3 were from contacts with a duration between 1 and 4 hours.

**Figure 2:**
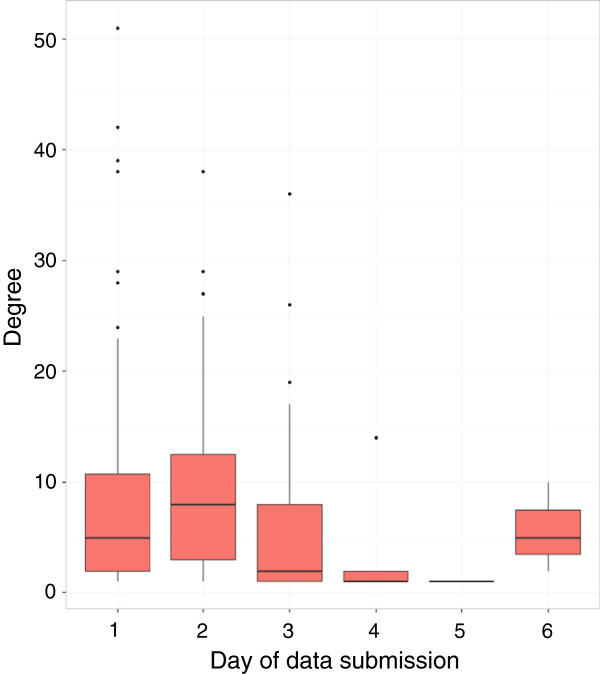
**Survey degree by day of data submission.** Box-and-whisker plots showing the reported degree distribution by day of data submission. Day 1 refers to the first day of data collection, March 14.

Most survey participants - 154 or 60.2% - submitted contact reports on the first day of data collection (March 14), and the degree distribution had a mean of 8.0. On the second day of data collection, 31 (12.1%) participants submitted contact reports; the mean degree was 10.4. On the third day, 61 (23.8%) participants submitted contact reports; the mean degree was 5.3. Ten additional participants submitted reports on the following days with a mean degree of 3.8. The degree distributions for the different report submission days are shown in Figure 2.

The motes detected a total of 13972 unique contacts. The vast majority of these contacts (11698 contacts or 83.7%) had an accumulated duration of less than 5 minutes; 1334 (9.5%) lasted 5 to 15 minutes, 899 (6.4%) 15 to 60 minutes, 38 (0.3%) 1 to 4 hours, and 3 (< 0.1%) longer than 4 hours.

### Nodal degree comparisons

**Figure 3:**
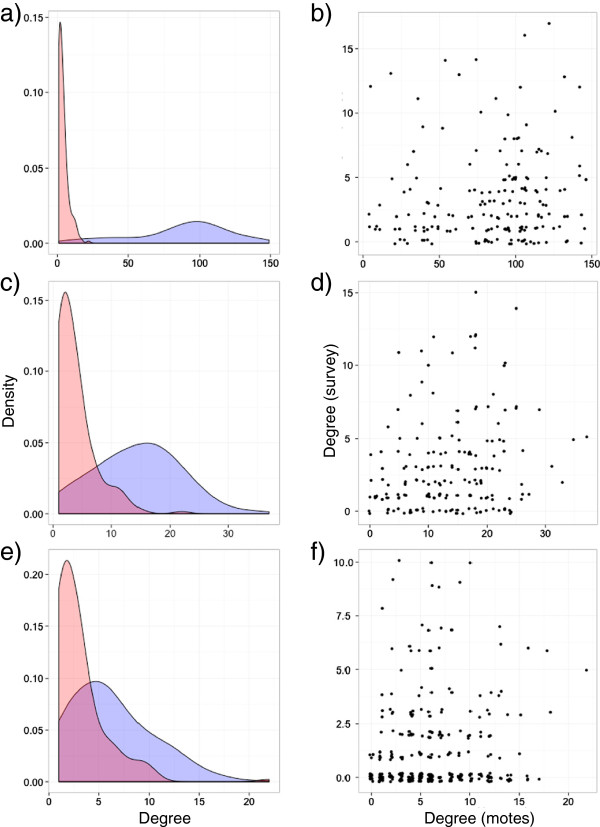
**Mote versus survey degree.** Left column **(a, c, e)**: kernel density estimates for survey (red) and mote (blue) degree distributions. Right column **(b, d, f)**: jittered scatterplots of the nodal degrees as measured by the mote study versus the nodal degrees as measured by the survey study. 1st row **(a and b)**: all contacts included; 2nd row **(c and d)**: only contacts longer than 5 minutes included; 3rd row **(e and f)**: only contacts longer than 15 minutes included.

The scale of the mote-based nodal degree distribution was approximately one order of magnitude higher than the scale of the survey-based nodal degree distribution when all contact durations were included in the analysis (Figure 3a). The scale of the mote-based degree distribution moved closer to the scale of the survey-based distribution when contacts with short durations were successively excluded from the analysis (Figures 3c and e). Further, there was no statistically significant correlation (at an alpha level of 0.05) between nodal degrees measured by the motes compared to nodal degrees measured by the web-based contact survey when all contacts were included (Figure 3b; τ = 0.097, p = 0.067), whereas we could detect significant, but rather weak correlation (i) when contacts shorter than 5 minutes were excluded (Figure 3d; τ = 0.142, p = 0.008), and (ii) when contacts shorter than 15 minutes were excluded (Figure 3f; τ = 0.206, p = 0.000).

### Average survey reporting probabilities

In total, 133 (23.5%) web-based survey contacts had concordant reports (i.e., the contact was reported by both participants) and 432 (76.5%) contacts were reported by only one study participant, but not by the other. Furthermore, 536 (3.5%; see Table 1: 15523 contacts in total – 14893 mote-recorded, but not reported – 94 reported, but not mote-recorded) survey contact reports corresponded to a mote-recorded contact and 14893 (96.5%) mote-recorded contacts were not reported by the respective participant. Table 2 shows a cross-tabulation for concordant and discordant records only from the survey data. Table 1 shows the distribution of all concordant and discordant records of survey and mote data across the five contact duration categories. Reporting probabilities, *P*, reporting probabilities conditional on mote detection, *P*_*s*_, and proportion of differences in mote and survey data due to underreporting, $\left(N_{1'}^{\mathrm{sm}}\;-\;N_{1}^{\mathrm{sm}}\right)/\;N_{3}^{\mathrm{sm}}$, are shown for different contact durations and different networks in Table 3.

**Table 1:** Cross-tabulation of contact data from motes and surveys

	**Mote measurements**						
**Survey reports**	**Not detected**	**< 5 min**	**5 - 15 min**	**15 - 60 min**	**1 - 4 h**	**> 4 h**	**Row Totals**
**Not reported**	**unknown** (n/a)	**12764** (85.7%)	**1309** (8.8%)	**802** (5.4%)	**16** (0.1%)	**2** (0.0%)	**14893** (100.0%)
	(n/a)	(98.8%)	(91.2%)	(78.2%)	(34.8%)	(100.0%)	(95.9%)
**< 5 min**	**22** (27.5%)	**37** (46.3%)	**10** (12.5%)	**11** (13.8%)	**0** (0.0%)	**0** (0.0%)	**80** (100.0%)
	(23.4%)	(0.3%)	(0.7%)	(1.1%)	(0.0%)	(0.0%)	(0.5%)
**5 - 15 min**	**31** (19.6%)	**49** (31.0%)	**36** (22.8%)	**38** (24.1%)	**4** (2.5%)	**0** (0.0%)	**158** (100.0%)
	(33.0%)	(0.4%)	(2.5%)	(3.7%)	(8.7%)	(0.0%)	(1.0%)
**15 - 60 min**	**35** (10.7%)	**64** (19.6%)	**66** (20.2%)	**140** (42.9%)	**21** (6.4%)	**0** (0.0%)	**326** (100.0%)
	(37.2%)	(0.5%)	(4.6%)	(13.6%)	(45.7%)	(0.0%)	(2.1%)
**1 - 4 h**	**6** (9.4%)	**6** (9.4%)	**13** (20.3%)	**34** (53.1%)	**5** (7.8%)	**0** (0.0%)	**64** (100.0%)
	(6.4%)	(0.0%)	(0.9%)	(3.3%)	(10.9%)	(0.0%)	(0.4%)
**> 4 h**	**0** (0.0%)	**0** (0.0%)	**1** (50.0%)	**1** (50.0%)	**0** (0.0%)	**0** (0.0%)	**2** (100.0%)
	(0.0%)	(0.0%)	(0.1%)	(0.1%)	(0.0%)	(0.0%)	(0.0%)
**Column Totals**	**94** (0.6%)	**12920** (83.2%)	**1435** (9.2%)	**1026** (6.6%)	**46** (0.3%)	**2** (0.0%)	**15523** (100.0%)
	(100.0%)	(100.0%)	(100.0%)	(100.0%)	(100.0%)	(100.0%)	(100.0%)

Columns give the number of contacts detected by motes by five duration categories and also the number of contacts that were not detected by the motes, but reported by a participant. Rows give the number of contacts reported by survey participants and also the number of contacts that were detected by the motes, but were not reported by the respective participant. Each percentage within a cell represents percentage of the row (right) and column totals (lower).

**Table 2:** Cross-tabulation of pairs of contact reports from the contact survey

	**Reported duration: higher value**					
**Reported duration: lower value**	**< 5 min**	**5 - 15 min**	**15 - 60 min**	**1 - 4 h**	**> 4 h**	**Row totals**
**Not reported**	**78** (18.1%) (98.7%)	**116** (26.9%) (89.2%)	**214** (49.5%) (75.6%)	**24** (5.6%) (34.8%)	**0** (0.0%) (0.0%)	**432** (100.0%) (76.5%)
**< 5 min**	**1** (12.5%) (1.3%)	**4** (50.0%) (3.1%)	**1** (12.5%) (0.4%)	**2** (25.0%) (2.9%)	**0** (0.0%) (0.0%)	**8** (100.0%) (1.4%)
**5 - 15 min**		**10** (21.3%) (7.7%)	**29** (61.7%) (10.2%)	**7** (14.9%) (10.1%)	**1** (2.1%) (25.0%)	**47** (100.0%) (8.3%)
**15 - 60 min**			**39** (62.9%) (13.8%)	**21** (33.9%) (30.4%)	**2** (3.2%) (50.0%)	**62** (100.0%) (11.0%)
**1 - 4 h**				**15** (93.8%) (21.7%)	**1** (6.3%) (25.0%)	**16** (100.0%) (2.8%)
**> 4 h**					**0** (n/a)	**0** (n/a)
					(0.0%)	(0.0%)
**Column totals**	**79** (14.0%) (100.0%)	**130** (23.0%) (100.0%)	**283** (50.1%) (100.0%)	**69** (12.2%) (100.0%)	**4** (0.7%) (100.0%)	**565** (100.0%) (100.0%)

Cell frequencies represent the reports by contact duration for pairs of participants. That is, participant A’s and participant B’s reports about their contact with each other comprise a single observation in the table. Columns give the higher contact duration report, and rows show the lower report. If a contact was reported by only one participant in a pair, then the lower duration value is ‘not reported’. Each percentage within a cell represents percentage of the row (right) and column totals (lower).

**Table 3:** Reporting probabilities and underreporting

**Network**	**Quantity**	**< 5 min**	**5 - 15 min**	**15 - 60 min**	**1 - 4 h**
All individuals	*P*	2.5%	19.4%	39.2%	78.9%
	*P*_*s*_	1.2%	8.8%	21.8%	65.2%
	$\left(N_{1'}^{\mathrm{sm}}\;-\;N_{1}^{\mathrm{sm}}\right)/\;N_{1}^{\mathrm{sm}}$	47.7%	40.0%	43.3%	50.0%
Degree > 1	*P*	3.5%	26.3%	48.1%	85.1%
	*P*_*s*_	1.5%	11.2%	27.0%	69.0%
	$\left(N_{1'}^{\mathrm{sm}}\;-\;N_{1}^{\mathrm{sm}}\right)/\;N_{3}^{\mathrm{sm}}$	42.4%	35.4%	40.0%	39.2%
Degree > 2	*P*	4.0%	28.9%	50.8%	85.7%
	*P*_*s*_	1.6%	12.4%	29.0%	73.0%
	$\left(N_{1'}^{\mathrm{sm}}\;-\;N_{1}^{\mathrm{sm}}\right)/\;N_{3}^{\mathrm{sm}}$	39.9%	34.9%	39.5%	45.0%
Degree > 3	*P*	4.3%	32.0%	55.8%	84.4%
	*P*_*s*_	1.8%	13.5%	31.5%	73.5%
	$\left(N_{1'}^{\mathrm{sm}}\;-\;N_{1}^{\mathrm{sm}}\right)/\;N_{3}^{\mathrm{sm}}$	40.8%	33.0%	36.4%	51.1%
Degree > 4	*P*	4.9%	27.7%	56.3%	88.5%
	*P*_*s*_	1.9%	14.2%	33.5%	75.0%
	$\left(N_{1'}^{\mathrm{sm}}\;-\;N_{1}^{\mathrm{sm}}\right)/\;N_{3}^{\mathrm{sm}}$	36.9%	43.2%	39.1%	39.0%
Smieszek et al. [38]	*P*	49.0%	81.0%	89.0%	95.2%

Reporting probabilities of (i) survey reporting, *P*, (ii) reporting probabilities conditional on mote detection, *P*_*s*_, and (iii) proportion of differences in mote and survey data due to underreporting, $\left(N_{1'}^{\mathrm{sm}}\;-\;N_{1}^{\mathrm{sm}}\right)/\;N_{3}^{\mathrm{sm}}$. Percentages for *P*, *P*_*s*_, and $\left(N_{1'}^{\mathrm{sm}}\;-\;N_{1}^{\mathrm{sm}}\right)/\;N_{3}^{\mathrm{sm}}$ were calculated for four predefined different contact duration categories and for five different networks (all participating individuals included; only participating individuals with a degree of more than 1, 2, 3, or 4 included), all compared to *P* values calculated from a previous study [38].

The main results are that (i) the reporting probability is considerably higher for contacts with a long duration than for contacts with a short duration, and that (ii) underreporting accounts for approximately one third to half of the difference between sensor-based and survey-based contact data.

### Individual differences in reporting quality

**Figure 4:**
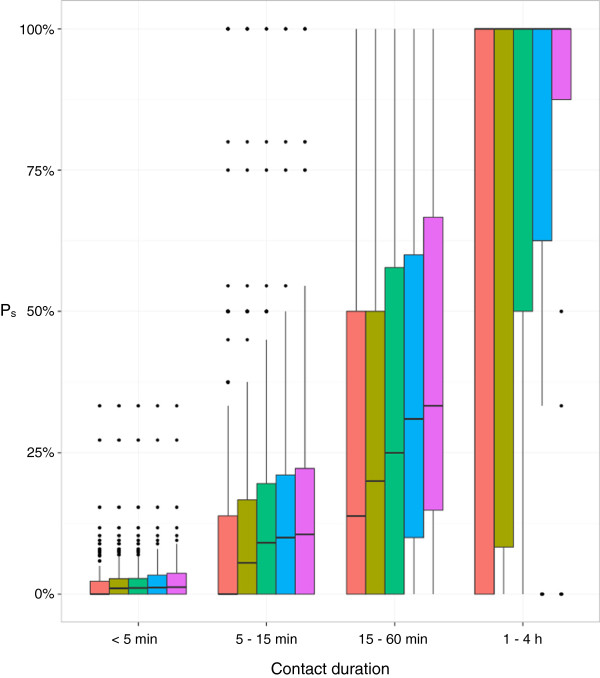
**Individual reporting probabilities.** Distributions of *P*_*s*_ (calculated for all individuals separately) rendered as box-and-whisker plots. *P*_*s*_ values are provided separately for the four duration categories < 5 minutes, 5 to 15 minutes, 15 to 60 minutes, and 1 to 4 hours. The red box-and-whisker plots show the distributions for all individuals, the olive ones for individuals who reported at least two contacts, the green ones for individuals who reported at least three contacts, the blue ones for individuals with at least four contacts, and the pink ones for individuals with at least five contacts. The whiskers extend from the median line to the highest/lowest value that is within 1.5 times the interquartile range.

Differences between mote and survey data at the individual participant level are illustrated in Figure 4. Consistent with average reporting probabilities, individual reporting probabilities increased with increasing contact duration. Excluding individuals with very few contact reports (i.e., degree lower than 2, 3, 4, or 5) amplified the overall reporting quality considerably. The probability to report a contact of ≤ 5 minutes duration that was detected by a pair of motes was very small: the median reporting probability for all participants was zero and even the 3rd quartile was close to zero. Only two participants achieved reporting probabilities of more than 25% for the less than 5 minute contact duration category. In contrast, the median reporting probability for the contacts that lasted longer than one hour was 100%.

When the study population was divided into a female and a male group, clear differences in the individual reporting probabilities between these groups appeared: While the mean reporting probability of females was 2.2% in the less than 5 minute contact duration category, we estimated only 1.1% for the male participants (p = 0.020, one sided test). In the category of 5 to 15 minutes, we estimated the mean reporting probability of female participants to be 12.4% and that of males 6.9% (p = 0.026). For 15 to 60 minutes, we estimated 30.9% for females and 18.7% for males (p = 0.006), and for 1 to 4 hours, we estimated 76.4% for females and 46.4% for males (p = 0.036).

Splitting the study population into different age groups did not result in a clear pattern.

## Discussion

Self-reported contact surveys and contact diaries have frequently been used for measuring contact networks relevant for the spread of infectious diseases [25-30]. More recently, technological advances have permitted the use of motes to record high resolution contact data [8,31-34]. For the first time, we compare these two methods for measuring contact networks in an identical setting. Overall, we found little congruence in recorded contact data between the two methods.

### Interpretation of the results

A comparison of the nodal degrees from both methods revealed no statistically significant correlation for all contacts and only weak correlation for contacts of higher duration (Figure 3b, d, f). Furthermore, the empirical degree distribution differed substantially between the two methods (Figure 3a, c, e). Since many studies rely on the nodal degree to fit models of infectious disease spread [7,10,25,43], this finding indicates that survey- and mote-derived degree data cannot be used interchangeably to inform infectious disease models, and it raises questions about the appropriateness of at least one of the two methods for collecting epidemiologically relevant contact data.

Consistent with previous contact survey research [38], we found that reporting probabilities increased with higher contact duration. In general, persons are typically more likely to recall and report contacts of longer duration than short interactions. Nevertheless, our web-based contact survey reporting probability estimates were considerably lower overall from both unfiltered and filtered network data, compared to earlier findings [38] (see Table 3).

Underreporting is a significant problem in reconstructing contact networks from survey data [23,38,44,45]. Therefore, we were interested in calculating the extent to which survey underreporting contributed to observed differences between the survey and mote data sets. We first looked at the proportion of differences when all participants were included in the analysis across all of the predefined contact durations (Table 3). We found that the proportion of the differences between the two methods caused by underreporting ranged from 40.0% to 50.0%. Excluding participants who reported very few contacts decreased the observed differences between the two methods related to underreporting (33.0% to 51.1% for individuals with a degree larger than three). This finding reflects the effect of individual participant differences in reporting quality.

To analyze the reporting differences between the survey and the mote datasets at the individual level, we calculated the fraction of a participant’s mote-detected contacts that was also reported by the respective participant (Figure 4). Consistent with the average reporting probabilities, we found that individual reporting probabilities increased with increasing contact duration, and that excluding individuals with very few contact reports improved the overall reporting quality considerably. We further found that female participants tended to report contacts more accurately than male participants. These data show how differences in individual participant survey reporting quality can contribute substantially to the difference between mote and survey data sets.

The overall reporting quality differs vastly among studies. For example, Smieszek et al. [38] reported 65.1% of all contacts had concordant reports, and Read et al. [23,46] reported that 30.2% of the contacts had concordant reports, both of which are higher than the reporting quality observed in this study (23.5% concordant reports, see Table 2). There are some plausible factors that may explain these observed differences. In particular, the study population and setting could have had important effects on the likelihood of reporting a contact. For example, Read et al. [46] obtained contact data from the students and staff at the University of Warwick, and Smieszek et al. [38] obtained contact data from members of three research groups at ETH Zurich, whereas the data presented here were obtained from students, teachers, and other staff at a US high school. First, it is reasonable to assume that it is cognitively more demanding to recall the many more and rather short contacts that are likely to occur at high school, than the fewer contacts likely to occur in a university research setting. Second, the motivation to contribute to a scientific study might be higher among members of a university than members of a high school.

### Appropriateness of contact definitions

Survey underreporting is highlighted as a reason for the observed differences between the contact data collected by the surveys and motes in this study (Table 3). However, even if survey reporting was perfect (e.g., participants were more motivated to complete the survey, and/or tried harder to remember all contacts, even those of shorter duration) surveys and motes would be unlikely to record exactly the same contact data. The reason for this is that the definition of a contact differs between the two methods: motes measured all face-to-face collocation events within a maximum distance of two meters, while the survey contact definition included only conversational contact that occurred at a maximum distance of two arm lengths. Thus, the contact definition underlying the mote measurements is more inclusive than the survey’s definition, and, hence, we would always expect more contacts detected by motes than reported using contact surveys or diaries.

The extent to which different contact definitions are meaningful for infectious disease transmission depends on the specific pathogen’s modes of transmission. There are four modes of transmission for respiratory infections: (i) droplet transmission (produced from the respiratory tract and expelled by various processes such as talking, sneezing, or coughing, and are at least 60 μm in diameter [40]), (ii) aerosol transmission (aerosolized particles of a small diameter that stay suspended in the indoor air), (iii) transmission through direct, physical contact, or, (iv) indirectly, through fomites [23]. The role of each of these for, e.g., influenza transmission is currently unclear [47-54]. For the purpose of this study, we were interested in droplet transmission, since there is evidence that close contacts are an important factor in the droplet transmission of many respiratory infections [55,56].

Droplet transmission, however, depends on various parameters such as droplet size and shape, the velocity with which the droplets are expelled, the viscosity of the droplets, as well as the temperature and humidity of the ambient air [40,57,58]. While droplets generated by breathing travel less than one meter, coughing can expel droplets that can be carried more than two meters away, and droplets produced by sneezing can potentially travel more than six meters [40]. Contact studies that relied solely on surveys could fail to record infectious contacts (e.g., via coughing and sneezing) if participants were further than a typical conversation distance apart. On the other hand, motes can be programmed to record interactions up to a specific distance, allowing motes to record all close proximity events regardless of whether any of the involved participants talked, sneezed, coughed, or did any other activity resulting in elevated levels of expelled droplets. Consequently, a contact study that relied solely on motes could result in an over-recording of events irrelevant for the spread of infectious respiratory disease.

Finally, transmission probability is a continuous function of the distance between the infectious and the susceptible individual, rather than a step function. Hence, imposing any specific cut-off will not result in an accurate representation of potentially contagious contacts. In essence, further empirical studies are necessary to test different contact definitions to assess which ones best explain the spread of infections in specific host-pathogen systems. These tests could include motes that are sensitive to changes in breathing, coughing and sneezing.

### Limitations of the study

We report contact data collected from a single school day at one US high school, limiting the generalizability of our findings. Comparing the participation rate of this study with other, methodologically similar studies is difficult, because many of those other contact survey studies used different strategies to obtain study participants, such as convenience samples, cohorts or quota sampling, and often did not report the number of people that participated, compared to the total number approached [e.g., [25,29,30,37,46]. A comparison with three other contact network studies at schools (which all differed in methodology) indicates that the participation rate in our study might have been lower than could be expected [27,59,60]. Reasons for lower participation might have included the overall burden of the entire study, which involved more components than reported in this paper [39], in addition to dynamics within the school (students or staff unwilling to participate if other peers did not participate).

Since other contact survey studies have shown substantial differences in reporting quality [23], the poor reporting quality we observed in this study may not occur elsewhere, including other high school settings. In addition to the retrospective design of our survey, a minor part of the underreporting in the surveys might have been caused by the design of our web-based survey: participants had to press an “add another contact” button for every additional contact they wanted to report. While the web-based survey was found to be easy to use and convenient in informal pre-tests, it cannot be ruled out that this design prevented users to report all contacts. Nevertheless, our research revealed that contact survey and mote measurements of exactly the same setting can result in almost unrelated contact networks, which poses questions about the appropriateness of at least one of the two methods.

Additionally, the method we used to estimate underreporting of contacts was based on four simplifying assumptions (for details see Smieszek et al. [38]): that (i) the probability of reporting a contact, *P*, depended solely on the contact duration; (ii) reports of a specific contact were stochastically independent; (iii) since the true duration of a contact was not known, the higher value was assumed to be true; (iv) incongruent contact reports were only due to under- and never due to over-reporting (i.e., we believe that the reporting participant was right). While these assumptions were certainly violated in some cases, they were essential to estimate underreporting given the data available from this study.

Finally, we were unable to match all partners reported in the web-based contact survey unambiguously to participants in the mote study (see “Linking survey and sensor data” subsection). One reason for such non-matchable reports is that individuals attempted to report a contact that actually took place, but did not report contact partners’ names correctly. If reported names were too different from actual names, matching was not possible, despite referring to the same contact event. However, even if we assume that all non-matchable contact reports would refer to a contact that actually took place, the results would only change slightly: The *P*_*s*_ for the unfiltered data would still be 1.2% for the < 5 minutes contacts, it would increase from 8.8% to 9.2% for the 5 to 15 minutes contacts, from 21.8% to 23.3% for the 15 to 60 minutes contacts, and from 65.2% to 67.3% for the 1 to 4 hour contacts.

## Conclusions

Results of our study suggest that sensor- and survey-based contact data cannot be used interchangeably for modeling infectious disease dynamics for all settings and all age groups. In the context of previous methodological studies, we have come to the following conclusions:

First, contact surveys are very flexible and can be designed to collect data in various settings. They are easy to design and do not require substantial technological skills on the part of the researcher. However, since reporting quality varies vastly between different settings and, likely, between different age groups, underreporting of short-duration contacts can be a very serious issue due to the potential relevancy for acute respiratory disease transmission. It is almost impossible to distinguish whether differences in individuals’ reported contact patterns are true differences, or whether they are a result of differing individual capabilities and levels of motivation. Furthermore, surveys are unlikely to be very useful in efforts to collect data on very young children or illiterate populations, who may not be able to complete contact surveys.

Second, validation on the level of individual transmission events is still lacking for both survey- and sensor-based approaches of data collection. While there have been attempts to validate empirical contact data on a population level [7,10,43], there is still no proof that such contact data could explain concrete transmission paths in a population. Validation on the level of individual transmission events is important for demonstrating that measured contacts are valid proxies for potentially contagious situations. Such validation could also assist in refining data collection methods for generating contact networks that best account for infectious disease transmission.

Third, while sensor-based approaches for measuring epidemiologically relevant contact networks are objective and substantially more reliable, new sensors need to be developed that overcome the disadvantages of the current generation of sensors. Ideally, new sensors will have the capability of distinguishing various close proximity events of different epidemiological importance (e.g., assessing whether the person is talking, coughing, sneezing, or none of these) and will be able to capture contact with partners who are not equipped with sensors.

## Abbreviations

CPR: Close proximity record; ID: Identification number; PDA: Personal digital assistant.

## Competing interests

The authors declare that they have no competing interests.

## Authors’ contributions

TS, VCB, and MS contributed to the conception and design of this study. VCB, IS, and MS collected the data. TS performed the data cleaning and the analyses. TS, VCB, JJR, HG, AU, and MS contributed to the interpretation of the data and analysis results. TS and VCB drafted the manuscript, and IS, JJR, HG, AU, and MS revised the manuscript draft. All authors read and approved the final manuscript.

## Authors' information

Disclaimer: The opinions expressed by authors contributing to this article do not necessarily reflect the opinions of the Centers for Disease Control and Prevention or the institutions with which the authors are affiliated.

## Pre-publication history

The pre-publication history for this paper can be accessed here:

http://www.biomedcentral.com/1471-2334/14/136/prepub
